# Notes of the Week

**Published:** 1921-06-18

**Authors:** 


### NOTES OF THE WEEK.

### A HUMAN DOCUMENT.

Following its practice for upwards of thirty years—the first Hospital Sunday Number was issued on June 15, 1889—The Hospital will issue a Special Hospital Sunday Supplement on June 25, the day before Hospital Sunday. To the devoted workers in hospitals, be they medical, nursing, or lay, it must seem almost an impossibility that amongst the general public there is an incredible amount of ignorance, even in these days when the hospital's work and influence have been brought so directly, through the war, into the life of most of us. Our Special Hospital Sunday Supplement is therefore addressed to the general public, and is designed to inform them on these aspects of life and their intimate relation with the well-being of the individual. It will contain an article by Mr. E. W. Morris, house governor of the London Hospital, on "A Day in a Hospital," describing a typical day in any large teaching hospital; articles on the appeal of Hospital Sunday and the work done for the hospitals by the Hospital Sunday Fund; "What the Hospitals Cost and Need"; the Social Service side of a hospital's work; Hospitals and Nurses; and figures showing a single year's roll-call of the sick. In addition, the hospitals of London have been invited each to tell their own story in our columns and to make their own appeal. The price of The Hospital with this Special Supplement will remain the same, viz. 2d., and orders should be placed in good time with the Manager, The Scientific Press, 28 and 29 Southampton Street, Strand, London, W.C. 2.

### THE CHILDREN AND HOSPITAL SUNDAY.

We are glad to see that the Metropolitan Hospital Sunday Fund is doing its best to interest the school-children in the cause of voluntary hospitals, and to-morrow, being the Sunday before Hospital Sunday itself, a printed story, by Miss Sibyl Longman, is to be distributed through the Sunday schools. This is a welcome new movement, which, apart from immediate results, will inculcate the habit of hospital support in the next and succeeding generations. The clergy are again giving useful help by processions and house-to-house collections, assistance doubly valuable at the present time when the difficulty of improving last year's success is obviously great. It seems, however, that the organisation has been developed, and thus that the movement is better able than ever to withstand the changes of the commercial world.

### THE CLOSED WARDS.

Lord Cave's Committee express no surprise that some of the hospitals have been obliged to reduce the volume of their work. "Indeed," says the report, "it cannot be expected that hospital managers will continue indefinitely to carry on their hospitals at a loss amounting, in some cases, to £1,000 a week." But at the London the loss is £2,000 a week and there is £85,000 owing to bankers and others. When one has borrowed to the hilt on free assets the only thing left is to ask the Charity Commissioners for permission to borrow on the security of the endowment fund; but this means serious loss of income, because a sinking fund has to be instituted. The position at a hospital which is forced to close beds is increased in difficulty by the obligations that every employer must feel towards the permanent staff. At the London, for instance, we understand the trained and valued staff is being retained in the hope that the wards will be reopened. This can be done in the holiday season with less loss than at other times of the year, but to close wards for any length of time and still to retain a full staff would mean the defeat of the object for which the wards were closed. The voluntary hospital position generally demands that the Government should announce their decision on the Cave recommendations without delay. The million pounds emergency grant is, after all, but an act of justice, as the Cave Report states that an actuarial investigation reveals the fact that in London alone the hospitals spent £530,000 more than they were paid on treating military patients.

### REJUVENESCENCE.

The death of a patient who had undergone Steinach's treatment for rejuvenescence has attracted much attention, and a good deal of nonsense has been written on the subject. Most writers in the lay press, including "a distinguished physician," have laboured under the impression that Steinach's treatment consists in the transplantation of a monkey's thyroid gland. Steinach's treatment, however, has nothing whatever to do with the thyroid gland: it consists of a stimulation, in various ways, of "hormones" which are especially associated with the phenomena of pubescence, and the results might perhaps more correctly be called "repubescence" than rejuvenescence. He has certainly established that certain internal secretions may be stimulated, and that some of the physiological activities of puberty may be restored; but it has yet to be proved that this restoration is in any way conducive to longevity, and it seems not unlikely that such anachronous physiological activity may ultimately hasten rather than delay old age. There is no proof, however, that the death of Steinach's patient was due to the treatment he had undergone.

### OIL FUEL AND COAL DIFFICULTIES.

The difficulty of heating operating-theatres has led to the postponement of operations at certain hospitals, and an interesting review of the position of the city hospitals in regard to coal has been published in the *Liverpool Courier*. Mr. A. Naldrett, secretary of the Royal Southern Hospital, announces that an oil plant has been ordered, one of the advantages of which will be a reduction of the staff and consequently of salaries. The Royal Infirmary is considering whether or not to resort to oil; two motor-lorries, though marked with the Red Cross and the name of the hospital, were turned back from the pithead at St. Helens by a crowd largely composed of women. The David Lewis Northern and the Stanley Hospitals are stated to have possessed good reserve, when the strike began, and Mr. H. Levey, secretary of the former, is reported to have experienced no difficulty. Whether or not oil can be as easily interfered with by transport workers as coal by its producers remains to be seen; and the policy favoured at present appears to be to heat one boiler by oil and to leave the other to be heated by coal when that shall be again available.

### FULL HUTS AND EMPTY WARDS.

A curious state of affairs exists in Camberwell, where a large slice of Ruskin Park has, for a number of years, been occupied by huts belonging to the 4th London General Hospital, which are still used for part of the residue of military patients. At the same time in King's College Hospital, which is within a few hundred yards of the huts, there are 200 beds vacant for lack of funds, and accommodation could also be obtained at St. Thomas's Hospital. The public would like their park back and King's College Hospital would no doubt be willing to enter into a reasonable financial arrangement for the care of the soldiers from the huts. Could they be removed at once, the park could be prepared for use next summer for the public of this growing neighbourhood.

### SUBSCRIPTIONS FROM LOCAL AUTHORITIES.

The Finance Committee of the Royal Victoria Infirmary, Newcastle-upon-Tyne, foresees an income for 1921 of £73,000 and an expenditure of £112,000. The income can maintain barely two-thirds of the present number of beds. In the search for new sources of income the Finance Committee remarks that £10,000 a year might be raised from in-patients' contributions, and perhaps some reduction of work or some contribution might be arranged from the out-patients. The committee also suggests that local bodies and corporations might give payments in return for the hospital's services to those injured in street accidents and fires. The duty of the approved societies is also emphasised, in which connection the committee urges the necessity for "an efficient registration department." The committee regards this of "first-rate importance." The public is also to be informed of the seriousness of the position. In our view the overwhelming obligation rests upon the approved societies. Since more than one of these has already agreed to the principle of repayment to the hospitals, a conference should be held and a general agreed policy be promptly decided.

### VENEREAL DISEASE AMONGST SEAMEN.

The campaign which was initiated by the National Council for Combating Venereal Disease, at the first meeting of the League of Nations last year, to enable members of the mercantile marine of all countries to receive adequate treatment for venereal disease, is now receiving active support. At the North European Conference on Venereal Diseases, which was recently held at Copenhagen, the interests of seamen were particularly advocated by the English delegates, and the Conference approved of a detailed scheme providing for the free treatment of seafarers' of all countries. The medical, social, and international difficulties of such a scheme are very great, but it is confidently anticipated that they will shortly be conquered, as the seafarers' organisations are giving a large measure of support to this important question, and it is also receiving the influential assistance of the National Council and similar bodies in other countries.

### OPPONENTS OF HOSPITAL SATURDAY.

On the anniversary of Hospital Saturday in Manchester and Salford, Mr. Fred Knowles, Secretary of the Fund, gave an interesting statement to the *Manchester City News* of the difficulties with which the Fund has to contend in Manchester. It is not the coal strike, nor the present unemployment, which he emphasises, but the action of certain employers who, Mr. Knowles remarks, "will not allow any approach to their employees." The reason for this attitude is not stated, and Mr. Knowles would do well to make it plain. "We are not allowed," he went on, "to put up notices in many workshops and offices as we would like," and he added that very much more might be done if this prohibition were removed. Unfortunately Mr. Knowles' remarks, which were well worth making, were tucked away in the middle of a column, the heading of which bore no indication of the interesting fact below. Hospital Saturday is a co-operative movement which is too well established to need defence. But evidently it has opponents among the Manchester employers, and their obstruction, and the reasons for it, can hardly be too widely known.

### AN EFFECT OF SMOKING IN THE WARDS.

A correspondent who has lately been treated in three hospitals in London writes to us to complain of the stuffiness of the wards during the recent spell of hot weather. He says that he has had the greatest difficulty to keep the windows open, and that the stuffiness of the wards was aggravated by the smoking of the patients. Himself a smoker, he can be acquitted of a fad in this respect, and his complaint is worth noticing because the general permission to smoke does mean that wards will become unbearably stuffy and are certain to smell of stale tobacco if the windows are not much wider open than they used to be. The fetish of draughts afflicts patients no less than other people; and we hope that our readers will test the ventilation of their own hospital wards by asking themselves the next time that they enter them whether they smell of stale tobacco. "Even on the hottest day," our correspondent writes, "I had the greatest difficulty in getting the windows open near me, lest the other patients should feel a draught, though the ward is less than half full and I am at the far end of it."

